# Challenges and Strategies in Interviewing People With Acquired Brain Injury and Communication Impairment in Qualitative Interview Studies: A Secondary Analysis of Interview Data

**DOI:** 10.1111/jocn.70437

**Published:** 2026-07-12

**Authors:** Henrietta Gustavsson, Eva Jangland, Lena Nyholm

**Affiliations:** ^1^ Department of Medical Sciences, Neurosurgery Uppsala University Uppsala Sweden; ^2^ Department of Surgical Sciences, Nursing Research Uppsala University Uppsala Sweden

## Abstract

**Aims:**

To explore challenges involved in designing and conducting qualitative interviews with people with communication impairment due to acquired brain injury, as well as analysing data from such interviews, and to map strategies used to facilitate participation.

**Design:**

Secondary analysis of qualitative interview data.

**Methods:**

A secondary qualitative content analysis was conducted using data from a previous multiple‐case interview study on daily life without cranial bone protection. Data comprised interviews with patients with acute severe brain injury, family members and staff, as well as logbook notes and field notes incorporated into transcripts.

**Results:**

Findings are presented as a timeline and thematic categories, highlighting challenges related to planning, interviewing people with communication impairment, and interpreting fragmented or partly non‐verbal narratives. Strategies included flexible interview procedures, the use of logbooks for planning and analysis, and field notes to capture non‐verbal cues. Involvement of family members facilitated participation and interpretation.

**Conclusion:**

Despite challenges throughout planning, interviewing, and analysis, individuals with communication impairment due to acquired brain injury contributed valuable insights. Involving persons close to the patient enriched and confirmed patient narratives, while direct questioning and documentation of non‐verbal communication added depth and breadth that could not be gathered from other sources.

**Implications for the Profession and/or Patient Care:**

The findings provide practical guidance for researchers and clinicians seeking to include people with communication impairment in qualitative research.

**Impact:**

- What problem did the study address?
  ○ People with communication impairment after acquired brain injury are often excluded from qualitative interview research.
- What were the main findings?
  ○ Meaningful participation was facilitated through flexible interviewing, communication support, field notes, and involvement of family members.
- Where and on whom will the research have an impact?
  ○ The findings may support researchers and clinicians working with people with communication impairment following acquired brain injury.

**Reporting Method:**

COREQ.

**Patient or Public Contribution:**

No Patient or Public Contribution.

## What Does This Paper Contribute to the Wider Global Clinical Community?

- This paper shows that communication impairment and the need for communication support may affect the whole qualitative interview process, including recruitment, consent, planning, data generation, and analysis.
- It demonstrates how logbook notes, field notes, and case‐level triangulation can support interpretation when narratives are fragmented or partly non‐verbal.
- It highlights family members as complementary informants and communication supports, while retaining the patient's first‐person perspective as central.

## 1 Introduction

Communicative and/or cognitive impairment is a common sequela following acquired brain injury (ABI) and affects a large group of people. Despite the high prevalence of these impairments, people affected by them are often excluded from qualitative research and interview studies (Lloyd et al. 2006; Paterson and Scott‐Findlay 2002; Simmons‐Mackie and Lynch 2013). As a result, first‐hand perspectives and lived experiences are missing from the body of knowledge.

### 1.1 Communication Impairment Following ABI

Acquired brain injury is a term referring to any type of brain damage that occurs after birth, usually divided into traumatic and non‐traumatic injuries. Causes of non‐traumatic ABI can be vascular, such as stroke or haemorrhages, but also infections, tumours, and anoxia (Goldman et al. 2022). Globally, stroke is the second leading cause of death and the third leading cause of death and disability combined (Feigin et al. 2021). For admitted traumatic brain injury (TBI), the incidence rate in Europe is 262 per 100,000 (Peeters et al. 2015).

*Aphasia* is a common language disorder after ABI. For instance, poststroke aphasia affects one‐third (34%) of patients with acute stroke (Li et al. 2024). Aphasia can affect language comprehension in either or both the expression and reception of communication. Reading, writing, and gesturing are also often affected. The degree of expressive aphasia varies from mild, with occasional difficulties finding the right words, to total loss of verbal output (Sheppard and Sebastian 2020).

Furthermore, ABI can impair communication in other ways. *Cognitive communication disorder* is defined by American Speech Language Hearing Association (MacDonald and Wiseman‐Hakes 2010; Togher et al. 2023) as difficulty with any aspect of communication disrupted by cognition. Injuries affecting cognitive functions, such as attention, memory, organisation, problem‐solving, and executive functions, will affect verbal and non‐verbal communication. This means that speaking, listening, reading, writing, and social interaction might deteriorate. In practice, this can manifest as fragmented or disjointed speech and difficulties with abstract or hypothetical content, often further exacerbated by fatigue and accompanying motor impairments (Lloyd et al. 2006; Carlsson et al. 2007).

Thus, people with communication impairment due to ABI constitute a large, heterogeneous group. This heterogeneity highlights the importance of person‐centred and individually adapted approaches when conducting qualitative interviews, as communication needs and supportive strategies may differ substantially between individuals. The localisation and severity of the injury affect the grade and nature of any communication impairments. The term *communication impairment* will hereafter be used for any communication difficulties, whatever the cause or characteristics, including conditions such as aphasia and cognitive communication disorder.

A systematic search and review conducted by Shiggins et al. (2024) on inclusion of people with aphasia in stroke trials found that in a majority of included randomised controlled trials, 215 RCTs and 56 RCT protocols, the inclusion criteria lacked definitions concerning the participation of individuals with communication impairment. Examples of the definitions used were: the ability to follow directions or difficulty executing commands. Including individuals with communication impairment due to ABI in interview studies presents certain challenges. Barriers often arise from the perceived complexity of obtaining informed consent, as the impairments can hinder both expression and understanding (Lloyd et al. 2006; Carlsson et al. 2007). Informed consent, a concept central to all research with human participants (World Medical Association 2013), requires that participants be fully informed and their consent fully understood. Additionally, the research participant must be able to communicate their decision to the researcher. Furthermore, recall problems due to ABI might affect the ability to remember what has been consented to (Carlsson et al. 2007). These difficulties have sometimes led to the misguided protection of vulnerable patients through systemic exclusion (Shepherd 2020).

The most commonly used method for conducting qualitative studies is semi‐structured interviews (Simmons‐Mackie and Lynch 2013). People with communication impairment who are less articulate may struggle to provide so‐called richness in data, as open‐ended questions can result in limited or brief responses. Additionally, they may have difficulty articulating their thoughts and feelings and organising their responses (Lloyd et al. 2006). Communication impairment is also often accompanied by cognitive deficits of varying degrees, which might make the credibility of interview responses questionable (Lloyd et al. 2006). These challenges in data collection are a potential reason why such individuals are often excluded from qualitative interview studies, to simplify the research process (Lloyd et al. 2006; Wilson and Kim 2021). To include only cognitively well‐functioning people who are reflective and expressive in their communication results in an insufficient or skewed knowledge base for subgroups of patients (Lloyd et al. 2006; Paterson and Scott‐Findlay 2002; Shepherd et al. 2019).

To overcome these challenges, proxies, such as family members or caregivers, are often interviewed instead. Their input can facilitate communication with people who have communication impairment (Lloyd et al. 2006; Eisikovits and Koren 2010). Although their perspectives are valuable, they cannot replace the unique insider perspective of the people living with these conditions. Previous studies have also suggested that people with aphasia value the opportunity to have their own perspectives heard, even when communication during interviews is challenging (Dalemans et al. 2009; Prior et al. 2020). Qualitative research, which explores lived experiences and personal viewpoints, is flexible enough to capture facets of reality even in cases of communication impairment (Lloyd et al. 2006; Paterson and Scott‐Findlay 2002; Carlsson et al. 2007). This highlights the need to develop and adapt methods beyond traditional interviews to enhance participation (Simmons‐Mackie and Lynch 2013; Wilson and Kim 2021). Being denied the opportunity to participate in research is exclusive and stigmatising in itself (Shepherd et al. 2019). The latest Helsinki Declaration (World Medical Association 2013) emphasises that underrepresented groups should have appropriate access, and asking people about participation aligns with the ethical principle of autonomy.

Communication in qualitative interviews should not be understood solely as an individual capacity, but as interactional and shaped by contextual support. Previous research has shown that adapted communication strategies and skilled communication partners can facilitate meaningful participation for people with communication impairment (Wilson and Kim 2021; Dalemans et al. 2009; Simmons‐Mackie et al. 2016). Nevertheless, people with communication impairment remain underrepresented in interview studies, or their participation is insufficiently described because of assumed difficulties in obtaining rich and credible data (Shiggins et al. 2024; Wilson and Kim 2021; Menger et al. 2023). Existing studies also often provide limited detail regarding how communication challenges are managed during data collection, and clearer reporting of supportive adjustments has been recommended (Wilson and Kim 2021; Luck and Rose 2007). Furthermore, less is known about the practical and analytical challenges arising throughout the whole interview process, including recruitment, consent, planning, data generation, transcription, and analysis. Addressing this gap is important for developing robust knowledge to support person‐centered research and care.

Therefore, this secondary analysis aimed to explore the challenges involved in designing and conducting interviews with people with communication impairment due to acquired brain injury, as well as in analysing data from such interviews. Additionally, the study aimed to map strategies used to facilitate their participation.

## 2 Methods

### 2.1 Design

This study was a secondary analysis of qualitative data. The data originated from a previous multiple‐case study aiming to explore daily life without cranial bone protection while awaiting cranioplasty after decompressive craniectomy (Gustavsson et al. 2024). The primary study used semi‐structured interviews and yielded a substantial amount of data beyond the original research aim. After completion of the original study and finalisation of the primary analysis, a secondary analysis (Beck 2013) was conducted, focusing on challenges related to planning and conducting interviews with people with communication impairment following acquired brain injury, as well as challenges arising during analysis of such interview data. The secondary analysis also aimed to identify strategies that facilitated participation in qualitative interview research. The study follows the Consolidated Criteria for Reporting Qualitative Research (COREQ) for interviews and focus groups (Tong et al. 2007).

### 2.2 Participants

The participants were adults who had undergone decompressive craniectomy as a life‐saving procedure for acute severe brain injury. In addition, family members and staff members closely involved in the patients' daily lives participated. The groups will hereafter be referred to as patients, family members, and staff.

Before their initial injury, all patients were working and managed their daily life independently. Following injury, they were sedated and treated with mechanical ventilation at a neuro‐intensive care unit and underwent decompressive craniectomy as a life‐saving procedure due to refractory intracranial hypertension. At the time of the interviews, 2–24 months had passed since the injury, and the patients were in different stages of recovery, awaiting reconstructive cranioplasty (see Table 1 for patient characteristics). All had been discharged from the hospital except one, receiving inpatient rehabilitation, and all patients experienced sequelae from their ABI. Their conditions ranged from managing daily life almost independently with occasional supervision from home care services to requiring continuous staff assistance with all daily activities. One patient remained in inpatient rehabilitation (Table 1).

**TABLE 1:** Characteristics of patients and interviews.

Diagnosis (*n*)	Traumatic brain injury	1
	Intracerebral haemorrhage	3
	Ischaemic stroke	1
	Subarachnoid haemorrhage	1
GCS‐M^a^ score at admission to neuro‐intensive care (median range)		5.5/1–6
Side of injury (*n*)	Left	4
	Right	2
Sex (*n*)	Female/male	4/2
Age in years	Median/range	54/33–64
Living situation at time of interview (*n*)	Living alone	2
	Cohabiting with family members or friends	3
	Inpatient rehabilitation	1
GOS‐E^b^ score at the time of interview	Median/range	4.5/1–6
Time from injury to interview	Months, median/range	11.5/2–24
Settings for patient interview (*n*)	Face‐to‐face	4
	Video call	1
	Telephone	1
	Family member present	2
Order of patient's and family member's interviews within case (*n*)	Patient interviewed first	2
	Family member interviewed first	4

^a^ Glasgow Coma Scale Motor (Teasdale and Jennett 1974).

^b^ Glasgow Outcome Scale‐Extended (Wilson et al. 1998).

As the patients had communication impairments that affected their ability to describe their lives, family members and staff members in regular contact with them were included to supplement and clarify their descriptions. In the original study, eight patients were approached through purposive sampling to ensure variation in diagnosis, neurological recovery, age, gender, and time passed since the injury. One patient declined participation. The original study therefore comprised seven patient cases involving patients, family members, and, in some cases, staff members. One of these cases involved a patient with a complete lack of communicative ability, including non‐verbal communication. In that case only family member and staff interviews were conducted. As the present secondary analysis focused on interviews involving people with communication impairment, that case was excluded from the analysis. The final sample for the present study therefore comprised six cases involving six patients, five family members, and two staff members (see Table 1 for patient characteristics). In total, 13 interviews were conducted.

All patients experienced some degree of cognitive communication impairment, such as recall problems, issues with focusing, fatigue, impaired insight, motor weakness, prosodic challenges, and problems narrating coherent, detailed stories. Four patients had aphasia—one had severe aphasia and was able to speak only single words, and three had mild aphasia, speaking almost fluently but struggling with finding words and recounting coherent narratives. Communication impairments were not formally classified using standardised speech and language assessments, as participant selection in the original study aimed to achieve variation in diagnosis, neurological recovery, and functional outcome following ABI rather than communicative characteristics specifically.

### 2.3 Setting

The patient interviews were conducted face‐to‐face (*n* = 4), in the patients' residences. Due to long distances and individual preferences, one interview was conducted via telephone and one via video call. In two cases, family members were present to provide support during the interviews (see Table 1).

### 2.4 Data Collection and Procedure

Potential participants were identified from the neurosurgical departments' waiting list by the first (H.G.) and last (L.N.) authors. All patients who met the inclusion criteria during the study's data collection period were contacted. Patients received a letter written in simple language informing them about the study on daily life without cranial bone protection and that they would soon be contacted about participation. About 2 weeks later, the last author, who had no previous professional relationship with the patients, called either the patient or a family member, depending on ease of contact, to confirm whether they could be approached by the researcher conducting the interviews. Upon agreement, the first author, a PhD student, provided detailed information about the study and determined suitability for inclusion. The first author had no prior contact with the included patients. Once patients had provided informed consent, or when capacity to provide fully informed consent was uncertain, participation was discussed in consultation with family members, with attention to patients' verbal and non‐verbal indications of willingness. The timing and setting of interviews were then arranged through phone calls, text messages, emails, or assistance from family members. The interview planning procedure was documented in a logbook, including details about contact, communication impairments, preferences for timing and location, and other relevant considerations, such as recovery stage, symptoms from the ABI, or complications from treatment. The logbook was included in the data analysed.

Interviews were primarily conducted individually, although family members were present during two patient interviews at the patients' request to provide support. Patient interviews included four face‐to‐face sessions, one video interview, and one phone interview (see Table 1). Family members were interviewed face‐to‐face in two cases and over the phone in three cases, whereas staff interviews were conducted face‐to‐face at the patients' residences. Each participant took part in one interview, and in cases where family members attended patient interviews, they had been interviewed individually beforehand. Interviews lasted between 5 and 60 min (patients: 8–60 min; family members: 10–60 min; staff: 5 min). The shorter interviews were primarily interviews with staff who had little to add about the phenomenon, and with one patient with severe expressive impairment who communicated mainly through gestures and single words. Longer interviews either generated more extensive accounts or were more fragmented, sometimes including matters beyond the interview focus. The interviews were conducted, audio‐recorded, and transcribed verbatim by the first author (H.G.). The transcripts were not returned to participants for comments or correction. This was due to the communicative and cognitive challenges in the patient group and because transcript return was not considered a meaningful form of participant validation in this population (Wilson and Kim 2021). However, understanding was checked during interviews through summarising and confirmatory questions.

As patients' communication abilities were not pre‐assessed, apart from input from family members, the interviews began with the broad question: ‘How is your daily life without cranial bone protection?’ Depending on the patient's capacity to respond, the question was narrowed and simplified by using short sentences and simple words, as well as body language. In addition, when necessary, guided by themes from family or staff interviews, a topic guide, or observations of the surrounding environment. The interview guide had originally been developed for the primary study on daily life without cranial bone protection and was flexibly adapted during interviews depending on participants' communicative abilities and responses.

Numerous descriptive field notes were taken during and immediately after the patient interviews, capturing non‐verbal communication such as gestures, pauses, facial expressions, emotions, and patient positioning. In cases where communication was affected by the loss of mimicry and gestures, observations regarding the environment, occasional use of aids, and reflections on verbal and non‐verbal communication were documented in the logbook. These field notes, along with explanatory notes from the logbook, were later incorporated into the transcripts. The logbooks also included information on the order of interviews within each case (see Figure 1). In summary, the data for analysis included logbook notes on planning and interview impressions, along with transcripts with incorporated field notes (see Table 2).

**FIGURE 1:**
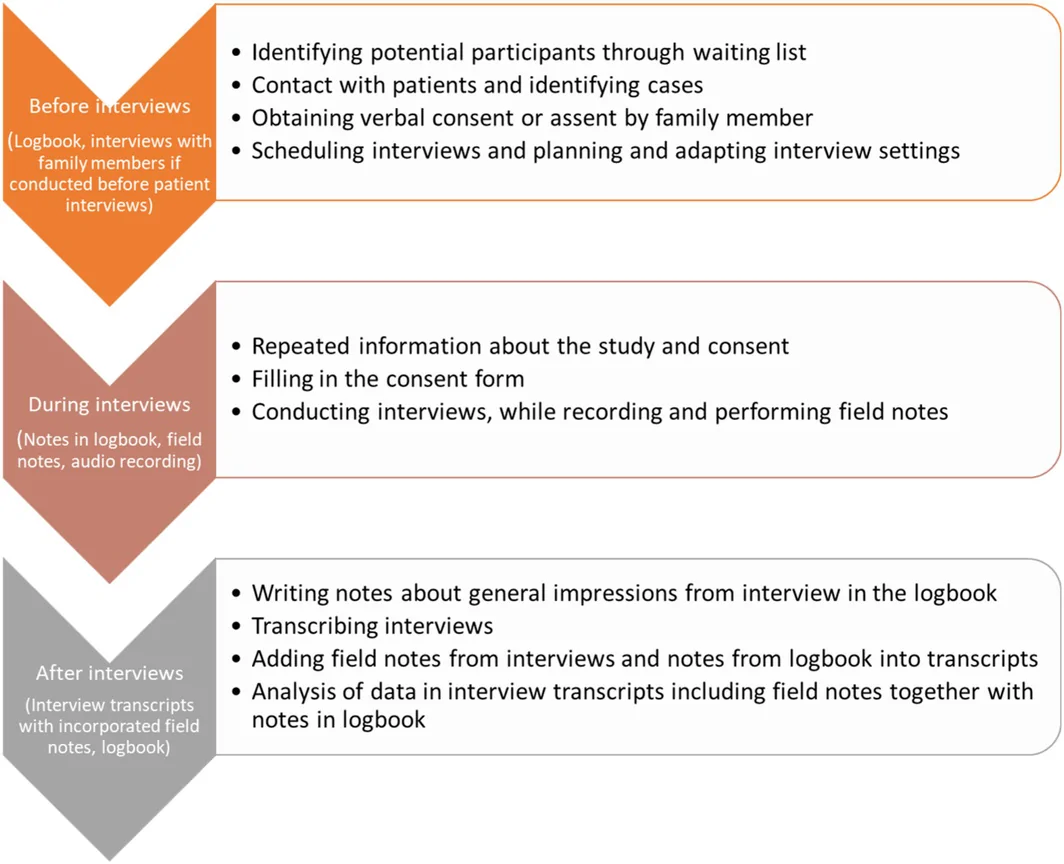
Flowchart of data collection and data sources over time. [Colour figure can be viewed at wileyonlinelibrary.com]

**TABLE 2:** Data sources within the three time periods (labels in *Result* section within brackets).

Before interviews	During interviews	After interviews
Transcripts from interviews with family members, if conducted before patient interviews, aiding the planning of patient interviews *(Interview with family member)*	Transcripts from interviews with family members and staff *(Interview with family member, Interview with staff)*	
	Transcripts from patient interviews with incorporated field notes of non‐verbal communication such as gestures, pauses, facial expressions, emotions, and patient positioning, and explanatory notes from the logbook *(Transcript, Transcript + Field notes)*	
Logbook containing Planning procedure (details about contact, preferences for timing, media, etc.) Recovery stage, symptoms from ABI, potential complications Order of interviews within case Nature of communication impairment General impressions from the interview situation, such as observations regarding the environment, occasional use of aids, and reflections on verbal and non‐verbal communication *(Logbook)*		

### 2.5 Data Analysis

The data underwent qualitative content analysis (Graneheim et al. 2017; Graneheim and Lundman 2004). This method was suitable for the study as it aims to explore variations in the phenomenon of interest, revealing similarities and differences in data. It can also be used to analyse text from interviews or narratives and observations. Data were analysed inductively, with the aim of exploring the challenges of designing and conducting interviews, analysing interview data, and mapping strategies to facilitate the participation of individuals with communication impairment in interview studies.

The analytical process can be said to have begun already during the transcription of the patient interviews. Field notes on gestures and other non‐verbal communication were incorporated into the transcripts, along with additional notes from the interviewer's recollection of the interview situation or logbook entries, to clarify any ambiguities understood from the context or to provide explanations for names or places mentioned. In instances where incorrect terms were used due to aphasia, interpretative comments were added as field notes to clarify the full meaning of these terms (see Figure 2). This facilitated the understanding of the transcripts for co‐authors, as the narratives were sometimes unstructured and fragmentary (see Supporting Information for an example from an interview with incorporated field notes). These data were not explicitly analysed in the original study by Gustavsson et al. (2024), but formed part of the data collection for this study. The transcribed interviews with incorporated field notes and the logbooks were then carefully reviewed by the first and last authors (H.G. and L.N.).

**FIGURE 2:**
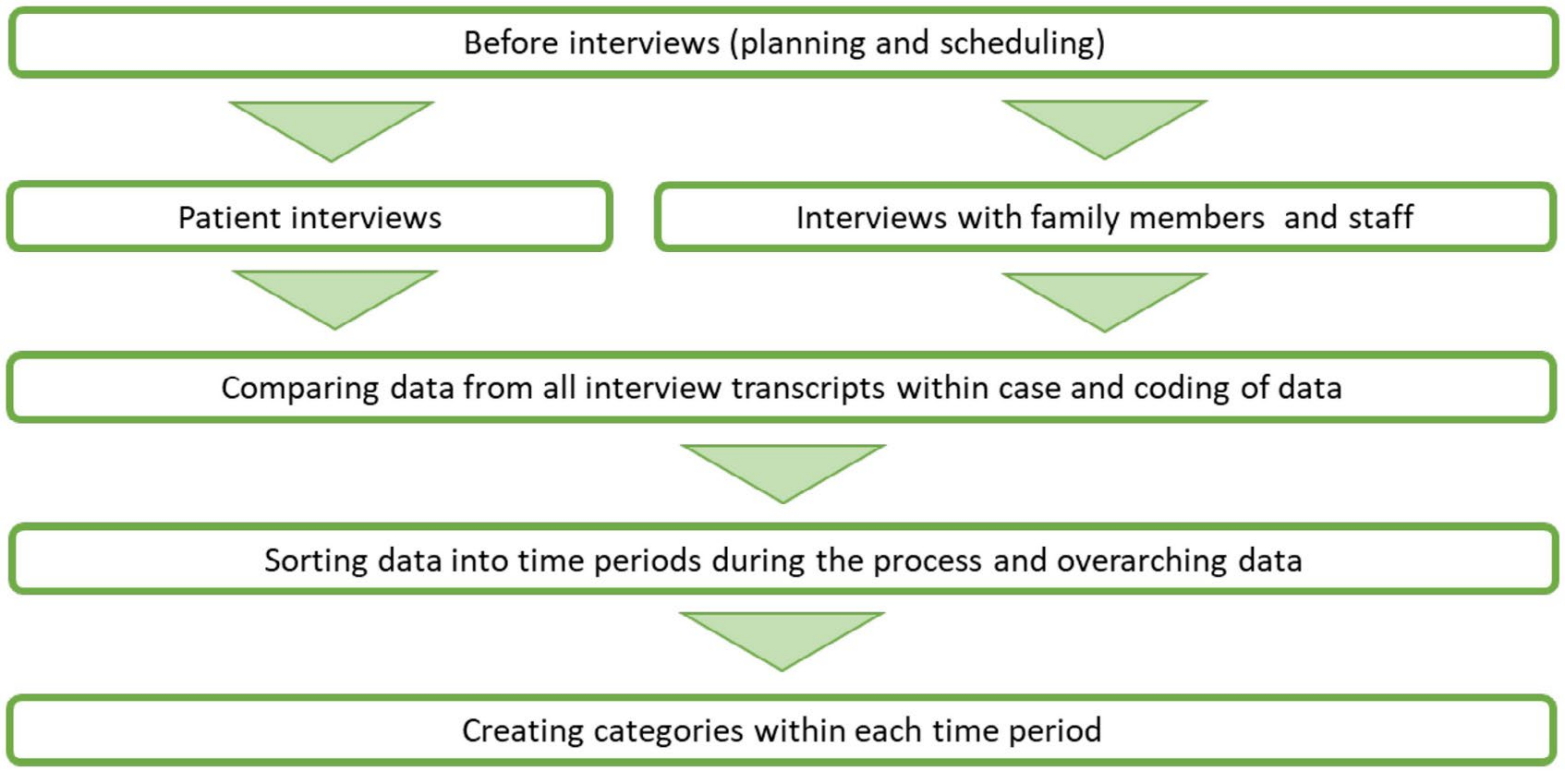
Flowchart of data collection and analysis process. [Colour figure can be viewed at wileyonlinelibrary.com]

Data were divided into meaning units (i.e., constellations of words or statements that related to the same central meaning), which were labelled with codes. The contents of the patient interviews where a family member was present were coded based on how the family member's presence aligned with the study's aim by facilitating and assisting during communication difficulties. The interviews with family members and staff were then compared with the patient interviews within each case to determine content congruence and how the narratives complemented each other. The intention was to identify whether certain contents appeared in only one of the interviews within a case, which, if so, was coded in accordance with the study's aim. H.G. and L.N. met and discussed the coding table several times during the process.

The codes were then sorted within three content areas (Graneheim and Lundman 2004), pertaining to different chronological time periods: before, during, and after the interviews. Although the temporal structure was introduced during analysis to organise the findings, the categories themselves were developed inductively from the data rather than predetermined prior to coding. Within each time period, the contents were expressed in one or several more comprehensive categories. However, during the analysis, data of a more overarching nature that spanned all three time periods—both challenges and strategies—were identified. Therefore, two categories were created that encompassed the entire timeline.

The first and last authors (H.G. and L.N.) met regularly throughout the analysis process to discuss the categorisation, and they compared the original texts and notes to ensure consistency in coding and categorisation. At the end of the process, the second author (E.J.) reviewed the analysis and contributed critical input. Revisions were made until a consensus was reached on the final result. During the analysis, the authors reflected on whether their backgrounds and experiences might have influenced the results (Graneheim et al. 2017) (see Table 3).

### 2.6 Ethical Considerations

This secondary analysis was conducted within the ethical approval granted for the original interview study by the Swedish Ethical Review Authority (2022‐02696‐01). Participants received written and verbal information about the study, including that participation was voluntary and that they could withdraw at any time without explanation. Information was provided in simple language. Although the information materials were not formally reviewed through patient and public involvement, they were adapted based on clinical experience and supplemented verbally and revisited as part of an ongoing consent process (Mayo and Wallhagen 2009) to support understanding for participants with communication impairment.

Because the interviews concerned the patients' own experiences, patients were asked to consent to their own participation as well as to interviews with family members and healthcare staff related to their case. In situations where capacity to provide fully informed consent was uncertain, participation was discussed in consultation with family members, alongside attention to patients' verbal and non‐verbal indications of willingness or dissent. No participant expressed dissent or wished to withdraw. Interview transcripts were pseudonymised during transcription, and only pseudonymised material was shared with co‐authors.

Ethical participation requires voluntariness and adherence to the World Medical Association Declaration of Helsinki (World Medical Association 2013). Inclusion of underrepresented groups may require adapted procedures to enable participation while safeguarding autonomy. Given the limited burden associated with interview participation and the importance of including first‐person perspectives from individuals with communication impairment, the study was considered ethically justified.

## 3 Findings

Various forms of challenges in collecting interview data occurred throughout the study process and are presented in categories within three descriptive time periods, ordered chronologically (see Table 4). Challenges and strategies overarching the entire process were identified, and the generated category was Communication challenges requiring continuous adaptation throughout the research process. Before interviews, the category of challenges was identified as *Interview planning requiring flexibility and support*. During interviews, the challenges identified are presented in the categories *Negotiating understanding of interview questions, Facilitating patients' responses*, and *Difficulties achieving mutual understanding*. After interviews, the category of challenges identified was *Grasping the patients' stories*. The strategies used to facilitate communication impairment during the process are described in italics, and data sources are given within brackets (also see Table 2). In the quotes, the field notes are written inside square brackets.

**TABLE 3:** Pre‐understanding among authors.

H.G.	Female, PhD student and certified critical care nurse. Has limited experience of conducting interview studies but extensive experience of clinical care of patients with brain injuries and communication impairment. Lacks formal education in communicating with individuals with aphasia. Has extensive knowledge of the patient group with their diagnoses and specific care and needs (18 years)
L.N.	Female, associate professor and certified critical care nurse. Possesses experience in qualitative research and conducting interview studies. Has significant expertise in clinical care of patients with brain injuries and communication impairment (28 years)
E.J.	Female, associate professor and specialist nurse in surgical care. Highly experienced in qualitative research and conducting interviews. Lacks specific experience with this patient population

**TABLE 4:** Overview of results.

Time period	Challenges	Strategies
Overarching	Communication challenges requiring continuous adaptation throughout the research process	*Flexibility in study procedures* *Field notes and logbook*
Before interviews	Interview planning requiring flexibility and support	*Help from family members and staff* *Verbal and written information* *Flexibility regarding timing and settings* *Informed consent in joint consultation with family members and as an ongoing process*
During interviews	Negotiating understanding of interview questions	*Targeted or direct questions* *Family members assisting*
	Facilitating patients' responses	*Field notes about non‐verbal communication* *Proposed words* *Family members assisting* *Targeted follow‐up questions*
	Difficulties achieving mutual understanding	*Targeted questions or summarising to clarify and confirm understanding* *Other interviews within case serving as guidance* *Family members assisting* *Adjusted timing to prevent fatigue*
After interviews	Grasping the patient' stories	*Inserting clarifying field notes during transcription* *Other interviews within case serving as guidance*

### 3.1 Overarching Challenges and Strategies

#### 3.1.1 Communication Challenges Requiring Continuous Adaptation Throughout the Research Process

It was found that the patients' ABI and associated communication impairment posed challenges that affected the entire study process, rather than being confined to a single time period. Aphasia, memory issues, cognitive difficulties, and challenges in recounting their story coherently and in detail were consistent obstacles throughout the process *(Logbook, Transcript + Field notes)*.

*Simply recording and transcribing the interviews would not have been sufficient to understand the patients' narratives; taking notes was necessary. The logbook notes facilitated the planning of interview situations by identifying potential communication impairments and useful strategies highlighted by family members. Documenting the planning process and any difficulties that arose served as a memory aid for the interviewer and supported the analysis of the interview material. The field notes taken during the interviews helped the co‐authors understand non‐verbal cues. They also aided the analysis by clarifying and providing context or details noted in the logbook or previously mentioned by the patient or others involved in the case (Logbook, Transcript + Field notes)*.

*Flexibility regarding the interview setting, practical execution of interviews, timing, etc., was crucial for the study's implementation, as deviations from the original plan were required to address the planning difficulties caused by the patients' ABIs. For example, a few patients wished for family members to be present during the interview, despite the original plan being for individual interviews (Logbook). Having the same person conduct both the planning and the interviews facilitated understanding, as it allowed for better recall of what had been said when interviews were fragmentary, or patients referred to previously mentioned topics without further explanation (Logbook, Transcript + Field notes)*.

### 3.2 Challenges and Strategies During the Interview Process

#### 3.2.1 Before the Interviews

##### 3.2.1.1 Interview Planning Requiring Flexibility and Support

Some patients had difficulties in planning their time, leading to uncertainties regarding the scheduling of appointments and situations where the agreed time was forgotten or rescheduled on short notice. Difficulties in handling technology made it challenging to establish contact and schedule appointments, as patients did not answer phone calls and only sporadically responded to text messages. This, combined with a sometimes short and brusque tone, was initially interpreted as unwillingness to participate. Some patients preferred not to be interviewed face‐to‐face, and some wanted to have family members present during the interview *(Logbook)*.

*To clarify and simplify matters for the patients, both verbal and written information in the form of letters, emails, and text messages was provided regarding the scheduled times. The home care staff, who assisted the patients in their daily activities, helped by clearly informing the patients of the scheduled meetings in their weekly plan (Logbook). Family members helped some patients with planning and booking the interviews (Logbook). The order of the interviews within the case became guiding; when a family member's interview was conducted before the patient interview, communication difficulties and planning problems could be highlighted, facilitating understanding and practical implementation. This confirmed that the dismissive expressions and difficulties in establishing contact were related to the patient's acquired difficulties and that they still wished to participate (Logbook, Interview with family member). For such reasons, a couple of the patient interviews were conducted over the phone or via video call instead of, as originally planned, face‐to‐face, and family members participated in two patient interviews (Logbook). One family member acted as a gatekeeper and requested that the interview with her be conducted before she facilitated contact with the patient, who would depend on her help to participate in the interview (Logbook, Interview with family member). Another patient called for a phone interview at a different time than scheduled, and the interview was then conducted immediately, in accordance with the patient's wishes (Logbook)*.

Informed consent was, in some cases, difficult to obtain because it was challenging to determine whether the information had been understood or remembered. Some patients also had physical difficulties filling out the form or did not understand how to complete it *(Logbook, Interview with family member)*.

Patient What should I write on it? Because I was a bit unsure about what should be there, whether I should say that someone else can come in and look at me […], or what it is?

*(Transcript; Patient 2)*

*In cases where the patient could not consent or where there was uncertainty regarding informed consent, patients were included in joint consultation with their family members. Family members also confirmed the patients' willingness to participate (Interview with family member, Logbook). One patient requested to review the interview topics in advance to become familiar with the material and then decide on participation (Logbook). The consent information was repeated during the interviews, and attention was paid to signs of possible dissent, especially in interviews where patients had difficulty expressing themselves (Transcript + Field notes)*.

#### 3.2.2 During the Interviews

##### 3.2.2.1 Negotiating Understanding of Interview Questions

Patients sometimes had difficulty understanding the questions posed by the interviewer, especially if the questions were open‐ended or broad. It could also be challenging for the patients to discern what the interviewer was seeking information about, leading them to respond to something other than what was asked. For example, they might describe stroke symptoms when the question specifically concerned the absence of cranial bone protection *(Transcript + Field notes)*.

*When patients could not answer broad questions, these were narrowed down to more targeted or direct questions to facilitate and clarify for the patients (Transcript)*.

Interviewer How do you sleep?

Patient Uh, how do you mean?

Interviewer Well, do you lie on this side or…?

Patient I lie…straight all the time.

*(Transcript; Patient 4)*

*In some cases, family members who were present asked confirming questions or pointed at the patient to aid understanding (Transcript + Field notes)*.

##### 3.2.2.2 Facilitating Patients' Responses

The patients sometimes had difficulties answering questions during the interviews, leading to ambiguities in their narratives. Aphasia can cause difficulties in finding individual words or incorrect use of words. The patient with the most severe aphasia could only communicate using single words and gestures. Other patients showed difficulties in the form of inability to narrate in detail, which led to short or terse answers *(Transcript + Field notes)*.

*When having difficulties finding words, patients reinforced their messages with facial expressions, tone of voice, and gestures. They pointed at themselves, objects, or around the room and showed how they performed certain tasks (Transcript + Field notes)*.

Patient And then I do like… *[shows how she rubs]*

Interviewer Yes, you rub?

Patient Yes.

*(Transcript + Field notes; Patient 1)*

*The interviewer proposed words or asked direct questions for clarification. Family members, when present, spontaneously helped by filling in words or clarifying based on their experience. Occasionally, patients turned to family members for help (Transcript + Field notes)*.

Patient Yes, I think so. Or? You have to help me here! *[turns to his wife and laughs]*

*(Transcript + Field notes; Patient 4)*

*Incorrect words were handled contextually, with alternative words suggested or just noted in field notes if they did not affect understanding (Transcript + Field notes). Despite word‐finding difficulties, context or previous discussion often made the patients' meaning clear, though clarifying field notes were sometimes required (Transcript + Field notes)*.

*In the case with severe aphasia, the family member narrated the patient's story during their interview. The patient then confirmed or denied parts of the story by answering questions based on the family member's narrative using single words, gestures, nods, or shakes of the head in response to direct questions or facial expressions to reinforce verbal responses (Transcript + Field notes). During this particular interview, the family member was present and occasionally asked relevant questions based on their experience of the patient's daily life and sometimes answered for the patient (Transcript + Field notes)*.

Interviewer Have I understood you correctly that […] That the skull bone is just a small thing among many other things…?

Patient *[nods]*

Interviewer The skull bone might not be the most difficult part?

Patient *[shakes head]*

Interviewer No…

Family member But the hardest part is probably speech, right?

Patient *[nods]*

*(Transcript + Field notes; Patient 5)*

*Brief responses were managed with targeted follow‐up questions to encourage elaboration. Facial expressions and tone of voice were used to reinforce the messages (Transcript + Field notes). Family members, when present, often clarified and expanded on the patients' narratives (Transcript)*.

Memory problems also affected the ability to answer questions and recount stories *(Transcript)*.

*In some cases, the interviewer's pre‐understanding guided the process when a patient had difficulty remembering events (Transcript, Logbook). Family members, when they were present, sometimes initiated or steered the conversation toward topics that complemented or confirmed statements or corrected inaccuracies in the patients' narratives (Transcript)*.

Family member And I think that was what made it difficult to put on the spectacles, because you were swollen there then.

Patient Yes […]

*(Transcript; Patient 4)*

##### 3.2.2.3 Difficulties Achieving Mutual Understanding

Fragmented narratives, lack of concentration, fatigue, and difficulty finding words or recounting led to the interviewer sometimes having difficulty understanding what the patient wanted to convey. This could manifest as answering something other than what was asked, forgetting the question, or changing the subject. Contexts, people, or places mentioned were not explained, and sometimes the interviewer suspected a lack of insight into the events in the narratives or noticed a discrepancy between what the patient and relatives or staff reported (*Transcript*).

*Targeted questions were used for clarification, follow‐up, or to confirm understanding in case of uncertainty (Interview transcript)*.

Interviewer […] maybe it's more about the energy levels, not the cranial bone…?

Patient Yes, yes, yes.

Interviewer …if I understand you correctly?

Patient Yes.

*(Transcript; Patient 1)*

*The interviewer could also summarise what was said to confirm the meaning, and in some cases the patient corrected or adjusted something that had been misunderstood (Transcript)*.

*The timing of each interview was adjusted based on when the patient was most alert (Logbook, Interview with family member), and the interviews with family members allowed the questions to be prioritised to focus specifically on the purpose, thereby conserving the patients' energy (Transcript, Logbook)*.

#### 3.2.3 After the Interviews

##### 3.2.3.1 Grasping the Patients' Stories

Difficulties in finding the right words or words being replaced by gestures made understanding challenging, especially for the co‐authors. This also occurred when stories were incoherent or fragmented and when patients spoke about events or people without any further explanation of the context. Memory problems and lack of insight could lead to direct inaccuracies, making it challenging to assess credibility *(Transcript + Field notes)*.

*All data sources, i.e., logbook, interview transcripts, and field notes, collectively formed the basis for the analysis. The process of understanding and grasping the essential points began already during transcription, where field notes were included in the transcripts*.

Patient But this one is too thick! *[shows the temple of the spectacles]* …to get it here now.

Interviewer Yes…oh, you mean it doesn't fit under the helmet?

*(Transcript + Field notes; Patient 2)*

*Patients sometimes mentioned something in passing, only to return to the topic later in the interview and explain it more thoroughly at that point. Incoherent narratives could sometimes be understood from the context or previously mentioned topics (e.g., people/names) in the transcripts and were then clarified with explanatory field notes to increase clarity for the co‐authors (Interview transcript + Field notes)*.

Patient that's probably both due to the disease and that it was more than…yeah, now, it's four…three years…since I, for example, uhm…requested a new…new…what's it called… Eye… *[talking about check‐ups with the optician and new spectacles]*

*(Transcript + Field notes; Patient 2)*

*Topics discussed during the planning phase occasionally reappeared in the interviews without clarification and were explained in the field notes to aid the co‐authors' comprehension (Logbook, Transcript + Field notes). Suspected inaccuracies in a patient's narrative, often due to lack of insight or memory issues, were analysed using the authors' and interviewer's pre‐understanding, together with the interviews conducted with family members and staff (Logbook, Transcript)*.

Interviewer Have they said anything about lying on the operated side?

Patient No, nothing.

The staff later confirmed in their interview that the patient had been informed of this.

*(Transcript + Field notes, Interview with staff; Patient 1)*

*If a family member was present, they occasionally questioned the patient's statements during the interview (Interview transcript). In cases of conflicting accounts, especially regarding experiences, the patient's first‐person perspective was prioritised, as their narrative formed the foundation of the analysis (Transcript + Field notes)*.

*Face‐to‐face interviews could sometimes validate the patients' statements, as the things they mentioned could also be observed (Transcript + Field notes). Listening to the recordings while reading the transcripts facilitated understanding, especially in cases where speech was less fluent*.

## 4 Discussion

### 4.1 Discussion of Results

This secondary analysis yielded several methodological insights related to conducting qualitative research involving people with communication impairment following ABI. The findings suggest that communication challenges and the need for adaptation may affect the entire research process rather than isolated interview moments, that contextual material such as logbook and field notes can serve as important resources for meaning‐making, and that flexible and partly co‐constructed interviewing, including involvement of family members when needed, may facilitate inclusion.

#### 4.1.1 Communication Challenges Requiring Continuous Adaptation Throughout the Research Process

A central finding was that communication impairment influenced not only the interview encounter itself, but also recruitment, consent procedures, scheduling, data generation, and analysis. However, these challenges were shaped not solely by impairment itself, but also by the adequacy of communication support and interviewer adaptations. This distinction is important to avoid locating barriers solely within the participant and instead recognise data generation as co‐constructed, shaped by communicative challenges as well as modifiable support factors. This aligns with previous work demonstrating that skilled communication partners can substantially facilitate participation for people with aphasia (Simmons‐Mackie et al. 2016).

Informed consent was one area where these challenges became particularly visible. Some patients forgot details of what they had consented to, a challenge also noted previously (Carlsson et al. 2007). However, treating consent as an ongoing process allowed repeated opportunities to confirm willingness to participate, while attention was paid to verbal and non‐verbal indications of dissent (Mayo and Wallhagen 2009). Information was therefore revisited repeatedly and adapted to the participant's communicative abilities to support understanding throughout the process. At times, patients appeared focused on reassuring the interviewer that everything was “okay” and that they were behaving as instructed, possibly reflecting deference or blurred boundaries between research and clinical care (Carlsson et al. 2007; Benson et al. 2021). The possibility that contextual factors, such as the interviewer's hospital attire in one interview, may have contributed to this underscores the importance of reflexivity.

Family members both facilitated and complicated inclusion. They often confirmed patients' willingness to participate but could also act as gatekeepers, a dual role described previously (Mayo and Wallhagen 2009). While involving family members may support inclusion and autonomy for people with communication impairment (Lloyd et al. 2006; Carlsson et al. 2007; Shepherd et al. 2019; Mayo and Wallhagen 2009), it may also introduce risks of influence, power imbalance, or undermining autonomy (Carlsson et al. 2007; Mayo and Wallhagen 2009). In our material, reliance on family support sometimes led to rescheduling, but also enabled participation that might otherwise have been lost.

#### 4.1.2 Logbook and Field Notes as Methodological Contribution

A key methodological finding was that logbook notes and field notes were not ancillary documentation but essential data sources in the interpretive process, particularly where verbal narratives were fragmented or supplemented by non‐verbal communication. Beyond facilitating repeated reading of transcripts, they aided interpretation of incomplete narratives, contextualised gestures and other non‐verbal expressions, and made analytic decisions more transparent. They were also necessary to provide co‐authors with a fuller understanding of the interview situations and to preserve contextual meanings that might otherwise have been lost when relying on transcripts alone. This may represent a methodological contribution by illustrating, in line with previous methodological discussions (Wilson and Kim 2021), how such materials can function not merely as documentation, but as active interpretive resources.

#### 4.1.3 Family Members as Support and Complementary Informants

Family members played a crucial role in the conduct of this study. Beyond contributing to planning, they functioned as communication support by providing background information and valuable context, suggesting relevant questions, and helping confirm or nuance patients' statements. This also allowed prioritisation of the most important questions in patient interviews, thereby conserving patients' energy, as discussed by Paterson and Scott‐Findlay (2002). When present during patient interviews, family members could provide a sense of security and sometimes support mutual understanding when communication difficulties arose, as previously highlighted by others (Lloyd et al. 2006; Wilson and Kim 2021; Dalemans et al. 2009; Howard et al. 2021).

However, balancing family members' involvement with preserving the patient perspective was challenging, as relatives sometimes unintentionally dominated conversations, for example, by “putting words in the patient's mouth” or redirecting focus away from the patient perspective, as noted elsewhere (Lloyd et al. 2006; Croteau et al. 2007; Philpin et al. 2005).

Within cases, patients, relatives, and staff were generally consistent regarding objective matters, whereas some divergence occurred concerning subjective experiences. Rather than invalidating patient narratives, such differences may reflect differing perspectives. Importantly, understanding often emerged at the level of the case through bringing together these different perspectives, rather than within any single interview alone, allowing triangulation across perspectives within cases. In this context, relatives may assess subjective experiences more negatively than patients themselves, a discrepancy also reported in previous proxy studies following stroke (Lapin et al. 2021; Oczkowski and O'Donnell 2010). An important distinction, therefore, is that the multiple case approach supported viewing family members and staff as complementary informants, not merely as proxy respondents, while retaining patients' first‐person perspectives as the primary interpretive anchor in analysis.

#### 4.1.4 Directed and Supportive Interviewing to Facilitate Inclusion

Because spontaneous open‐ended responding was often difficult, more directed and supportive interviewing was sometimes necessary to enable participation, consistent with Luck and Rose (2007) and literature on communication partner support (Simmons‐Mackie et al. 2016). Directed questioning appeared particularly useful where topics were broad, abstract, or memory‐dependent.

Our pre‐understanding and experience of engaging with individuals with ABI also informed how questions were formulated and adapted. The interview guide helped ensure that key topics were covered, particularly when broad, abstract, or memory‐dependent questions were difficult for participants to respond to.

Capturing non‐verbal communication was crucial. Audio recordings alone would not have been sufficient to support interpretation, as also suggested by Wilson and Kim (2021) and Luck and Rose (2007). Field notes documented gestures, facial expressions, pointing, and other communicative strategies used to convey meaning. Recordings were also listened to repeatedly when speech was unclear, a strategy also described by Philpin et al. (2005). Transparency regarding how such interpretations were made is important for credibility in studies involving communication impairment (Luck and Rose 2007).

Understanding was continuously checked during interviews through summarising and confirmatory questions, allowing participants opportunities to correct interpretations. Together with logbook notes, field notes, and multiple perspectives within cases, this supported the interpretive process. Rather than compromising qualitative interviewing, such support may be understood as an inclusion strategy.

#### 4.1.5 Flexible Adaptations, Including Interview Mode

Beyond adaptations within the interview interaction itself, flexibility in how interviews were arranged was also necessary to support participation. For some participants, remote or telephone interviews enabled participation that might otherwise not have been possible, although such modes reduced access to non‐verbal communication and could increase fatigue (Anglade et al. 2022; Topping et al. 2021). Although video recording has been recommended by others (Dalemans et al. 2009; Luck and Rose 2007), this was not feasible due to privacy and consent considerations. Flexibility also concerned timing, as interviews sometimes needed to be scheduled when participants were most alert. This flexibility also meant that interview conditions were not fully standardised across participants.

Similar flexible adaptations have been described by Howard et al. (2021). One participant requested interview topics beforehand, which proved helpful and has also been suggested by Wilson and Kim (2021) and Philpin et al. (2005). Email interviews may offer similar advantages (Howard et al. 2021; Egan et al. 2006), allowing participants to reflect without stress. Taken together, these adaptations suggest that an adapted interview, even if it departs from conventional qualitative ideals, may be more valuable than no interview at all when inclusion of participants with communication impairment is the goal.

### 4.2 Methodological Discussion

This study has methodological limitations that warrant discussion. Because communicative challenges and strategies were not the explicit focus during the original data collection, a more systematic sampling strategy might otherwise have been adopted to include a broader range of communication impairments and a more structured categorisation of challenges and strategies. However, recruiting participants based on diagnosis, recovery, and functional variation rather than communicative characteristics may also represent a strength, as the identified strategies appeared applicable across individuals with differing communicative profiles and levels of functioning. As the original study focused on daily life without cranial bone protection, the topic itself may also have shaped which communicative challenges became visible. More abstract or emotionally complex topics might have generated different communicative demands and required other adaptations. As Luck and Rose (2007) emphasise, aphasia is heterogeneous, and strategies may therefore vary across participants and contexts. The identified strategies may therefore also be relevant in qualitative interview contexts where researchers do not have specialist training in aphasia communication support.

A further limitation concerns interviewer influence and interpretation. Different responses might have been obtained with another interviewer or through repeated interviews. The use of more directive support increased the importance of transparency and verification. Consistent with Wilson and Kim (2021), understanding was continuously checked during interviews through summarising and confirmatory questions, functioning as an in‐interview form of verification. Although the researchers had extensive clinical experience of caring for patients with ABI, no speech and language therapist was involved in the planning or conduct of the interviews, which may be considered a limitation. Brief or fragmented responses may also increase the influence of interviewer interpretation, particularly when meaning must be inferred from context or supported through field notes and interactional cues. In addition, communicative abilities may fluctuate over time due to recovery processes, fatigue, and contextual factors, which may have influenced the interview interactions.

The interpretive use of multiple perspectives within cases may be seen as both a strength and a risk. While accounts from relatives and staff helped contextualise and sometimes corroborate patients' narratives, they could also potentially shape interpretation. We therefore did not treat consistency across sources as objective verification in a positivist sense, but rather used multiple perspectives to support interpretive understanding while retaining patients' first‐person perspectives as central. Logbook notes, field notes, and case‐level triangulation may also be understood as supporting credibility and confirmability, consistent with principles of trustworthiness in qualitative research (Elo et al. 2014).

The transcripts were not returned to participants, which may be considered a limitation. At the same time, returning transcripts alone may not have been the most meaningful form of participant validation in this population, particularly given communicative and cognitive challenges (Wilson and Kim 2021).

Dividing the findings into time periods facilitated readability; however, because the process itself was not always chronologically linear, this structuring may have simplified a more complex reality and entailed some risk of data loss in the analytic process. Although the findings are presented chronologically, the analysis itself remained inductive and iterative throughout the process.

This study does not claim exhaustive coverage of communicative challenges but offers methodological insights drawn from a particular set of interviews. Despite these limitations, the material was considered sufficient to address the research question.

### 4.3 Research Implications

When conducting interview studies with people with communication impairment due to ABI, flexibility and adaptation throughout the research process are essential. For clinical nursing and rehabilitation research, the findings highlight the importance of planning for communication support, fatigue, non‐verbal communication, and family involvement when including people with ABI in interview‐based research. Researchers and clinicians conducting interviews may benefit from increased awareness of communication support strategies and accessible interview procedures. Table 5 summarises methodological considerations identified in this study.

**TABLE 5:** Considerations when conducting interview studies with people with communication impairment.

Assess whether the chosen method aligns with your objectives and consider alternative ways to collect data Ensure that the same person handles scheduling, interviewing, and transcribing, as this establishes familiarity and facilitates trust and understanding Prioritise key questions to use interview time efficiently and conserve the patient's energy, or consider conducting multiple interviews Capture non‐verbal communication through methods such as notes, video recordings, or involving multiple interviewers, and be prepared to replay recordings multiple times during transcription to better understand unclear speech Have a thorough understanding of the patient group and put questions in a form that does not require a wordy response Consider incorporating support from family members, caregivers, or staff when needed to facilitate communication, interpretation, and contextual understanding

## 5 Conclusion

Despite the challenges posed by communication impairment affecting the entire study process, including planning, interviewing, and analysis, this study demonstrates that individuals with communication difficulties due to ABI can provide valuable insights in interview studies. Involving persons close to a patient complemented patient narratives, showing that descriptions were consistent within cases. Additionally, posing direct questions and documenting non‐verbal communication offered depth and breadth that could not be gathered from other sources. These findings contribute to the development of strategies for including this patient group in qualitative interview research. Such approaches may also enhance the understanding of individuals with communication impairment. By amplifying these individuals' voices, we contribute to building a stronger and more comprehensive evidence base.

## Funding

The authors have nothing to report.

## Conflicts of Interest

The authors declare no conflicts of interest.

## Supporting information


**Data S1:** An example of a patient interview transcript with incorporated field notes, illustrating how verbal narratives, non‐verbal communication, and contextual observations were documented and integrated into the analytic process.

**Data S2:** COREQ (COnsolidated criteria for REporting Qualitative research) Checklist.

## Data Availability

The data that support the findings of this study are available on request from the corresponding author. The data are not publicly available due to privacy or ethical restrictions.
