# Supplementary material for: Challenges and Strategies in Interviewing People With Acquired Brain Injury and Communication Impairment in Qualitative Interview Studies: A Secondary Analysis of Interview Data
Source: J Clin Nurs. 2026 Jul 12;35(11):5084–98. doi: 10.1111/jocn.70437 (PMC13629267; doi:10.1111/jocn.70437)
Supplement: Supplementary file 2 [file JOCN-35-5084-s002.docx]

**Supplementary Material**

(Example from a patient interview, with incorporated field notes in italics and within square brackets)

Interviewer: Yes. But what do you think now, about the fact that it’s taken so long? Do you mean that you’re losing hope?

Patient: Yes…yes, both that and, like, just a thing like… I want to… I feel that… And that’s probably both due to the disease and that it was more than…yeah, now, it’s four…three years…since I, for example, uhm…requested a new…new…what’s it called… Eye… *[talking about check-ups with the optician and new spectacles]*

Interviewer: Yeah.

Patient: Like, and improve it, and see if that’s necessary…

Interviewer: Yeah.

Patient: And that’s probably both because I’ve been without…or haven’t updated it for…yeah, it must be three…it must be four years now, that I’ve haven’t really…yeah, the last time before…yeah, then…then it looked fine.

Interviewer: Is it the optician that you…?

Patient: Yes, the optician. And about a year ago, then this happened in between as well and so…it’s been almost another year now…

Interviewer: Okay. Yes.

Patient: Or, not quite, but… So then… I think…or I feel that…there’s, like, something wrong, but if that’s just the disease…or also… I think it’s both.

Interviewer: Okay. With your eyesight…?

Patient: That I’ve gotten older as well.

Interviewer: Right. But do you feel that your eyesight has worsened? Did I understand that correctly?

Patient: Yes.

Interviewer: Yes.

Patient: Yes, and…and then you want to, like, get it…but then I don’t want to do it until this is gone, because it…

Interviewer: Yes.

Patient: Because this…how much will it…? Yeah. I’m not sure.

Interviewer: Are you thinking about the helmet?

Patient: Yes.

Interviewer: That it…gets in the way?

Patient: Yes. Because, for example, right now I have two pairs. I have two pairs, normally *[stands up and gets their spectacles]*. And one pair I never use anymore. These are like out… When I’m out driving *[shows the spectacles].*

Interviewer: Yes. Mhm… Right.
